# Development of Fluorescent Aptasensors Based on G-Quadruplex Quenching Ability for Ochratoxin A and Potassium Ions Detection

**DOI:** 10.3390/bios12060423

**Published:** 2022-06-16

**Authors:** Cheng Yang, Xiaolin Chu, Li Zeng, Amina Rhouati, Fathimath Abbas, Shengnan Cui, Daiqin Lin

**Affiliations:** 1 State Key Laboratory of Fine Chemicals, Department of Chemistry, School of Chemical Engineering, Dalian University of Technology, Dalian 116024, China; 21907036@mail.dlut.edu.cn (X.C.); ferthunabbas@mail.dlut.edu.cn (F.A.); cuishengnan@mail.dlut.edu.cn (S.C.); 2 Institute of Inspection and Testing for Industrial Products of Jiangxi General Institute of Testing, Nanchang 330052, China; zengli19890610@163.com; 3 Bioengineering Laboratory, Higher National School of Biotechnology, Constantine 25100, Algeria; amina.rhouati@gmail.com

**Keywords:** G-quadruplex, label-free, fluorescence aptasensor, photo-induced electron transfer

## Abstract

G-quadruplexes have received significant attention in aptasensing due to their structural polymorphisms and unique binding properties. In this work, we exploited the fluorescence-quenching properties of G-quadruplex to develop a simple, fast, and sensitive platform for fluorescence detection of ochratoxin A (OTA) and potassium ions (K^+^) with a label-free fluorophore and quencher strategy. The quenching ability of G-quadruplex was confirmed during the recognition process after the formation of the G-quadruplex structure and the quenching of the labeled fluorescein fluorophore (FAM). The fluorescence-quenching mechanism was studied by introducing specific ligands of G-quadruplex to enhance the quenching effect, to show that this phenomenon is due to photo-induced electron transfer. The proposed fluorescence sensor based on G-quadruplex quenching showed excellent selectivity with a low detection limit of 0.19 nM and 0.24 µM for OTA and K^+^, respectively. Moreover, we demonstrated that our detection method enables accurate concentration determination of real samples with the prospect of practical application. Therefore, G-quadruplexes can be excellent candidates as quenchers, and the strategy implemented in the study can be extended to an aptasensor with G-quadruplex.

## 1. Introduction

DNA is an important molecule responsible for storing and completing genetic information. It is well known that DNA can form different conformations under specific conditions, including single-stranded hairpins, double- or triple-stranded helices, and quadruplex structures [1,2,3]. The G-quadruplex is a unique secondary structure of DNA folding from a single-strand or several strands of DNA sequences rich in guanine with a highly ordered and diverse structure [4,5]. According to the direction of its DNA strands, it can be divided into parallel, antiparallel, and mixed structures [6]. The G-quadruplex structure mainly depends on the length of the DNA sequence, the orientation of the chain and loop, and the cation nature [7]. G-quadruplex has attracted the attention of relevant researchers, pushing them to develop various platforms based on G-quadruplex for the analysis and detection of a variety of substances, including metal ions [8,9], toxins [10,11,12], nucleic acids [13,14], and other small molecules.

According to previous reports, the fluorophore can be quenched at an appropriate distance using nucleotide bases by photo-induced electron transfer (PIET), especially between guanine bases [15,16,17]. Since G-quadruplex is a structure with a high accumulation of guanine and low oxidation potential [18], it can be ideally used as an electron donor. Therefore, resonance energy transfer or photo electron transfer may occur between G-quadruplex and the fluorophore, resulting in significant fluorescence-quenching induced by G-quadruplex. Previous studies have established the detection of lead and silver ions using fluorescence-quenching between G-quadruplex and HEX [19]. It has been demonstrated that the quenching between the G4/hemin complex and fluorescent dyes can be employed as a logic circuit platform [20]. Unfortunately, the exact mechanism of fluorescence-quenching caused by the G-quadruplex has not been widely accepted by the academic community.

Ochratoxin A (OTA) and potassium ions (K^+^) were used as model analytes to demonstrate that antiparallel and parallel G-quadruplex structures can act as quenchers for the label-free fluorescence probe, SYBR Green I (SGI). By replacing SGI with a fluorescent label FAM (fluorescein), we demonstrated that fluorescence-quenching was not caused by structural disassembly. However, this phenomenon was caused by the formed G-quadruplexes during the recognition process. In order to further investigate the quenching mechanism between G-quadruplex and fluorophore, we studied the fluorescence change in the system after the interaction between G-quadruplex and specific ligands of G-quadruplex, crystal violet (CV) for antiparallel G-quadruplex and hemin for parallel G-quadruplex, respectively. We found that adding the G-quadruplex specific ligand to the system leads to the accumulation and attachment of the ligand to the G-quadruplex, thus decreasing the fluorescence signal intensity. We believe that the fluorescence-quenching ability of the G-quadruplex is mainly due to PIET. Therefore, based on the excellent quenching ability of the G-quadruplex, we designed a simple label-free fluorophore and quencher fluorescence detection platform for OTA and K^+^ with good sensitivity and selectivity.

## 2. Experimental Section

### 2.1. Reagents and Chemicals

The sequence forming the antiparallel G-quadruplex is a new aptamer, which truncated five useless bases from the 3′ end of the original OTA aptamer (OTAapt36) to create a new blunt end aptamer (OTAapt31) [21]. OTA-A9 to OTA-A12 are the sequences to form a hybrid with an OTA aptamer. The parallel G-quadruplex sequence is 5′-GG GTG GGT GGG TGG G-3′, called G4. By adding complementary bases to the 3′ and 5′ ends of G4, we obtained a series of sequences with different bases, named G4, 1AG4, 3AG4, 5AG4, 7AG4, 9AG4, 11AG4, 13AG4, and 15AG4 (the parts that can form complementary sequences are underlined in the list shown in Table S1). The sequence that forms a hybrid with the parallel aptamer is 5′-CCC ACC CAC CCA CCC-3′, designated G4-C15. By shortening the bases at the 3′ end of C15, we designed a series of sequences with different bases, named G4-C11 to G4-C15. OTAapt31-FAM and 5AG4-FAM were the antiparallel and parallel G-quadruplex labeled with fluorescein (FAM) at the 3′ end. The nucleotide sequence is shown in Table S1.

All synthesized oligonucleotides were purchased from Shanghai Sangon Biotechnology Co. Ltd. (Shanghai, China). A stock solution of 100 µM for each oligonucleotide was prepared by dissolving them in ultra-pure water and then diluting the samples to the required concentrations before use. The concentrations were determined by measuring solution absorbance at 260 nm by the ReadMax 1900 Absorbance Microplate Reader. DNA stock solutions were stored at 4 °C before use. Sodium chloride (NaCl), potassium chloride (KCl), calcium chloride (CaCl_2_), magnesium chloride (MgCl_2_), ammonium chloride (NH_4_Cl), zinc chloride (ZnCl_2_), hydrochloric acid (HCl), methanol, Dimethyl sulfoxide (DMSO), Toluene (C_6_H_5_CH_3_), and dichloromethane (CH_2_Cl_2_) were purchased from Sinopharm Chemical Reagent Co., Ltd. (Beijing, China). Tris (hydroxymethyl) carbamate (Tris, C_4_H_11_NO_3_), SYBR Green I (SGI), and Tween 20 were purchased from BBI Life Sciences (Shanghai, China). Ochratoxin A (OTA), Ochratoxin B (OTB), Aflatoxin B1 (AFB1), Aflatoxin G1 (AFG1), Patulin, Fumonisin B1 (FB1), Zearalenone (ZEN), Deoxynivalenol (DON), crystal violet (CV), and hemin were purchased from Sigma-Aldrich (St. Louis, MO, USA). All the chemicals were at least analytical grade.

### 2.2. Apparatus

All solutions were prepared with ultra-pure water from the Millipore Direct-Q Pure Water Treatment System (Millipore Corporation, Burlington, MA, USA). All fluorescence measurements were performed at room temperature on a Safire II multi-detection micro-plate reader (Tecan, Switzerland). The fluorescent groups SGI and FAM provide fluorescence in the system. The excitation wavelength was 483 nm, and the emission wavelength was 525 nm. The UV Absorbance spectra were performed at room temperature on the ReadMax 1900 Absorbance Microplate Reader (Shanghai Flash Spectrum Biotechnology Co., Ltd., Shanghai, China).

### 2.3. Demonstration of the Mechanism of Fluorescence-Quenching

For the antiparallel G-quadruplexes system, different concentrations of OTA were mixed with 20 nM OTA aptamer (antiparallel G-quadruplex) in 10 mM Tris-HCl buffer (pH = 8.4) containing 0.3× SGI, 0.2% Tween 20 and 6.25 mM Ca^2+^. For parallel G-quadruplexes, different concentrations of K^+^ were mixed with 20 nM G-quadruplex (parallel) in 10 mM Tris-HCl buffer (pH = 8.4) with 0.3× SGI, 0.2% Tween 20. All solutions were incubated at room temperature for 20 minutes, and the fluorescence intensity was measured using a Safire II microplate reader. CV (antiparallel structure-specific ligand) and hemin (parallel structure-specific ligand) were added to the parallel and antiparallel G-quadruplex systems, and the fluorescence was measured after three minutes of incubation. All experiments were repeated three times independently.

### 2.4. Fluorescent Detection of OTA and K^+^

For OTA, 80 µL of different concentrations of OTA were mixed with 20 µL of 100 nM aptamer (antiparallel G-quadruplex) in a solution of 10 mM Tris-HCl buffer (pH = 8.4), with 1.5X SGI, 1.0% Tween 20 and 31.25 mM Ca^2+^. For K^+^, 80 µL of different concentrations of K^+^ were mixed with 20 µL of 100 nM parallel G-quadruplex in 10 mM Tris-HCl buffer (pH = 8.4), with 1.5× SGI, 1.0% Tween 20. All the test solutions were incubated at room temperature for 20 minutes, followed by the fluorescence measurement with a Safire II microplate reader. All experiments were repeated three times independently.

### 2.5. Specificity of the Aptasensor

To investigate the selectivity of the sensing method, we compared the fluorescence intensity changes induced by the two sensing systems with potential interfering toxins and ions, respectively. Under optimized conditions, OTB, AFB1, AFG1, FB1, patulin, ZEA, and DON were used as interfering substances for OTA detection. In parallel, NH_4_^+^, Na^+^, Ca^2+^, Mg^2+^, Zn^2+^, and Cd^2+^ were used as interfering substances for K^+^ detection. The fluorescence intensity change was detected according to the method described in Section 2.4. All experiments were repeated three times independently.

### 2.6. Detection of OTA and K^+^ in Real Samples

To verify the feasibility of OTA and K^+^ detection methods in real samples, red wine (purchased from local supermarkets) and goat serum were used as real samples. In the spike recovery experiment, 2.0 µL, 4.0 µL, and 8.0 µL of 10 µM OTA standard solution were added to 10 mL of red wine samples to obtain a series of spiked sample solutions. The spiked samples were pretreated using the extraction protocol described in our previous study [21]. The samples after pretreatment were detected according to the method described in Section 2.4, and the fluorescence intensity was recorded. Goat serum samples were diluted 1000-fold with Tris-HCl buffer (10 mM, pH 8.4). The corresponding concentrations of goat serum samples were calculated from the calibration curve and compared with the atomic absorption spectrometry (AAS) measurements. All experiments were repeated five times independently.

## 3. Results and Discussion

### 3.1. The Principle of Fluorescence Detection of OTA and Potassium Ions Based on G-Quadruplex Quenching

**Scheme 1:**
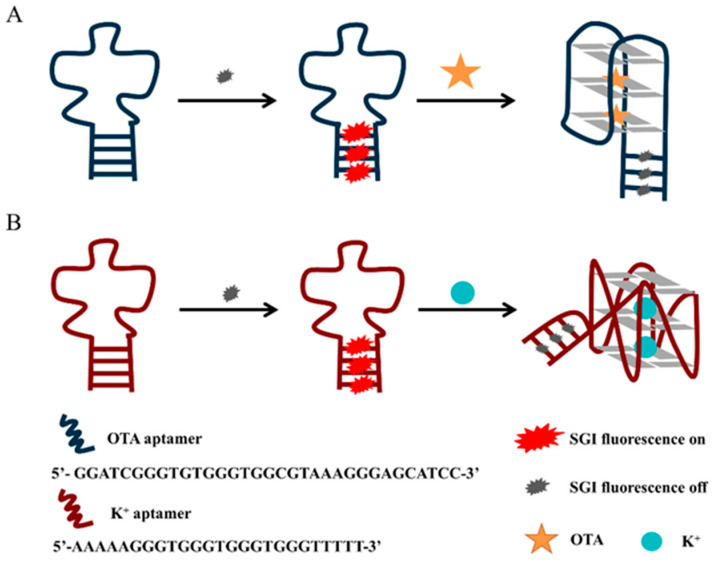
Schematic illustration of fluorescent aptasensors based on G-quadruplex quenching ability (**A**) for the detection of OTA (**B**) for the detection of K^+^.

**Figure 1:**
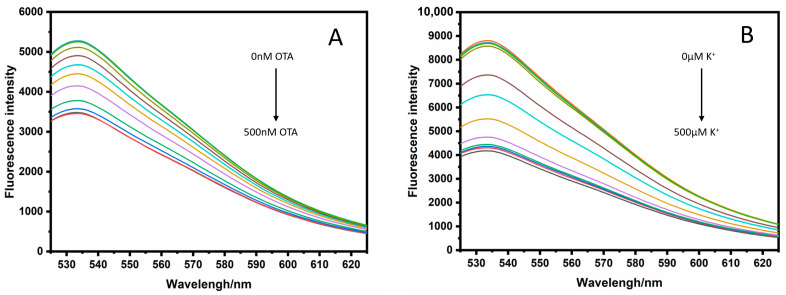
(**A**) Fluorescence emission spectra of unlabeled fluorescence antiparallel G-quadruplex with different concentrations of OTA (0, 0.48, 0.96, 1.95, 3.9, 7.8,15.6, 31.2, 72.5, 125, 250, 500 nM), (**B**) Fluorescence emission spectra of unlabeled fluorescence parallel G-quadruplex with different concentrations of K^+^ (0, 0.48, 0.96, 1.95, 3.9, 7.8,15.6, 31.2, 72.5, 125, 250, 500 µM).

Scheme 1 illustrates the principle of the proposed platform for the detection of OTA and K^+^. In solution, the fluorescent dye SGI in the free state can only emit weak fluorescence, but the fluorescence is significantly enhanced when SGI binds to double-stranded DNA. The complementary sequences at the 5′ and 3′ ends of the designed DNA sequence will hybridize, hence the intercalation of SGI into the duplex helix, resulting in fluorescence. The PIET process can occur between the G-quadruplex and the fluorescent dye embedded in the duplex helix under the induction of the target, resulting in a clear fluorescence-quenching phenomenon. Therefore, based on this principle, OTA and K^+^ can be quantitatively monitored in the solution. For an antiparallel G-quadruplex, we designed a new aptamer that truncated five useless bases from the 3′ end of the original OTA aptamer (OTAapt36) to create a new blunt end aptamer (OTAapt31) [21], which can effectively shorten the distance between fluorophore and quencher, enabling a detection that could not be achieved before. As shown in Figure 1A, it was observed that the system exhibited a significant fluorescence intensity at 525 nm in the absence of OTA, which was attributed to the binding of SGI to the duplex helix strand. The oligonucleotides originally in the random coil state were induced to form an antiparallel G-quadruplex structure by adding OTA. The increased number of the formed G-quadruplex structures leads to a more profound PIET phenomenon between the G-quadruplex and the intercalated SGI, where the fluorescence-quenching phenomenon is also more significant. For parallel G-quadruplexes, we designed oligonucleotides that can be induced to form parallel structures by K^+^. It can be easily seen that the parallel structure G-quadruplex formed under the induction of K^+^ has a specific quenching effect on the fluorescent dye embedded in the double-strand (Figure 1B).

### 3.2. Discussion on the Fluorescence-Quenching Mechanism

Considering that the SGI escape due to double-strand helix dissociation can also lead to fluorescence decrease, we investigated using FAM-labeled G-quadruplexes (OTAapt31-FAM and 5AG4-FAM). We replaced the label-free fluorophore with a labeled fluorophore (FAM) at the end of the aptamer. FAM has similar fluorescence characteristics to SGI. The FAM is directly labeled on the nucleic acid chain at a ratio of 1:1, and it does not detach from the labeled nucleic acid. So, the fluorescence intensity can visually reflect changes in the environment surrounding the FAM. The target (OTA or K^+^) induces the aptamer conformational change to a G-quadruplex structure (antiparallel or parallel). While the fluorescence intensity was still showing a downward trend with the increase of target (OTA or K^+^) concentration (Figure S3). It can be noted that the quenching effect of G-quadruplex was effective for labeled fluorophore, indicating that the quenching is because of the PIET mechanism instead of the dissociation of duplex strands.

The fluorescence intensity increased after adding the complementary sequences to the FAM-labeled antiparallel G-quadruplex system. The results showed that the hybridization of the complementary strands destroyed the structure of the G-quadruplex (Figure S4), resulting in the inhibition of fluorescence-quenching. A previous study depicted an aptasensor utilizing guanines as a quencher based on PIET [22]. However, the quencher is different from the current study. The FAM-labeled aptamers used in this research display strong fluorescence, although they were rich in guanine (G). With the addition of the target, the G-rich sequence formed a G-quadruplex to quench FAM. Taken together, this further confirms and verifies that the presence of the G-quadruplex is responsible for the quenching phenomenon.

The fluorescence-quenching mechanism can be divided into dynamic quenching and static quenching. The static quenching depends on forming a non-fluorescent ground state complex between the fluorescent molecule and the quencher [23], which is reflected in the absorption spectrum change of the fluorophore. However, the position and intensity of the absorption peak of the fluorescent molecules do not change in the presence of the G-quadruplex. The dynamic quenching comprises energy transfer [24] or electron transfer [25], which changes only the excitation spectrum of the fluorophore but not its absorption spectrum. The prerequisite for the occurrence of fluorescence resonance energy transfer is that both the fluorescent donor and acceptor molecules can fluoresce [26]. However, the G-quadruplex absorbs approximately 260 nm and shows very low fluorescence quantum yields [27].

In order to confirm the basic principle of the fluorescence-quenching of G-quadruplexes, we introduced the ligands of G-quadruplex, crystal violet (CV) for antiparallel G-quadruplex, and hemin for parallel G-quadruplex. Both complexes have a larger π-plane structure, and the binding sites of the complex and the G-quadruplex are on the outer G-tetrad.

**Scheme 2:**
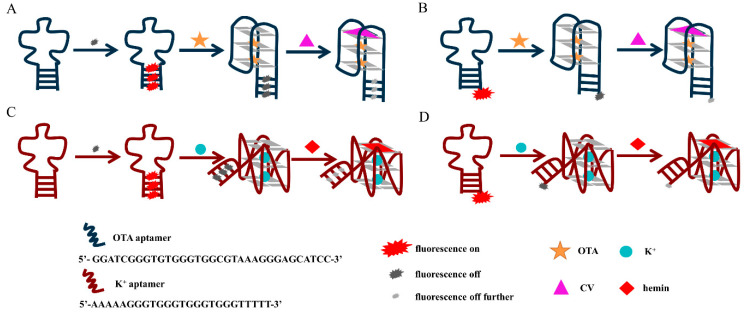
Schematic illustration of verifying the G-quadruplex quenching mechanism with the ligands of G-quadruplex (**A**) Antiparallel G-quadruplex unlabeled (**B**) Antiparallel G-quadruplex FAM-labeled (**C**) Parallel G-quadruplex unlabeled (**D**) Parallel G-quadruplex FAM-labeled.

**Figure 2:**
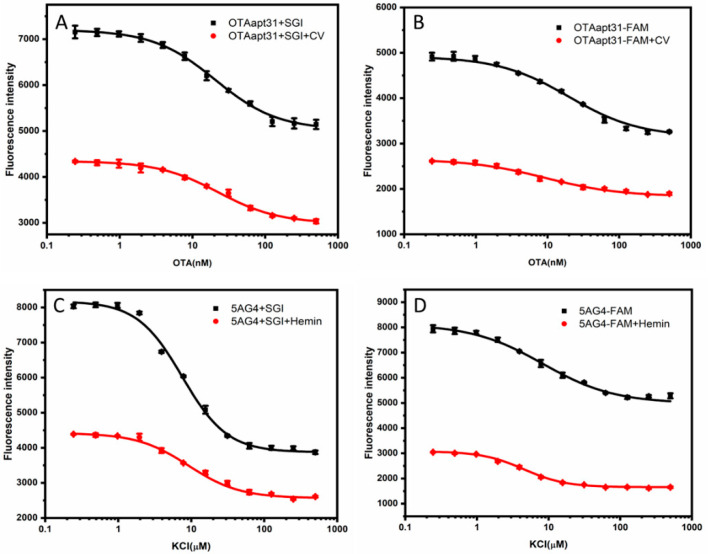
(**A**) Changes of SGI fluorescence response curves of antiparallel G-quadruplexes before and after adding CV at different OTA concentrations (**B**) Changes of FAM fluorescence response curves of antiparallel G-quadruplexes before and after adding CV at different OTA concentrations. (**C**) Changes of SGI fluorescence response curves of parallel G-quadruplexes before and after adding Hemin at different K^+^ concentrations (**D**) Changes of FAM fluorescence response curves of parallel G-quadruplexes before and after adding Hemin at different K^+^ concentrations. The error bar represents the standard deviations of the three parallel experiments.

CV is a triphenylmethane dye, and the fluorescence of CV bound to G-quadruplex with an antiparallel structure was significantly higher than that of G-quadruplex with a parallel structure in solution because the end loops of the antiparallel structure protect the bound CV from the solvent. It has been shown that the binding of the antiparallel G-quadruplexes to CV usually occurs in the outer G-quadrant [28]. Therefore, the principle of the verification process is shown in Scheme 2A,B, and the results are shown in Figure 2A,B. After the combination of CV with the antiparallel-G quadruplex, the fluorescence in the system was more significantly quenched. These results indicate that introducing CV increases the electron cloud density of the formed G-quadruplex/CV complex, and electrons are more easily transitioned from the G-quadruplex to the ground state positions released by the fluorophore. As a result, it is difficult for the electrons in the excited state of the fluorophore to return to the ground state, which manifests as a substantial decrease in the overall fluorescence intensity.

For the parallel G-quadruplex system, we explored the mechanism of fluorophore quenching by introducing hemin, one of the common G-quadruplex ligands which bind to the parallel G-quadruplex with strong affinity. The parallel G-quadruplex and hemin binding usually occurs at the 3′end of G-tetrad [29]. The principle of the verification process is shown in Scheme 2C,D, and the results are shown in Figure 2C,D. After adding hemin, the quenching phenomenon of fluorescence is more significant. This phenomenon can be attributed to the G-quadruplex/hemin complex, which provides more electrons. These electrons occupy the ground state position of the fluorophore, and more electrons in the fluorophore are excited, which is manifested in a substantial decrease in the overall fluorescence intensity.

The results demonstrate that the ligands add to an antiparallel or parallel G-quadruplex structure induce an increase in the electron cloud density of the structure, thus exhibiting a dramatic quenching of fluorescence. Moreover, we can confirm that the quenching of the fluorophore by the G-quadruplex was mainly caused by electron transfer. When the electrons of the fluorophore jump from the ground state to the excited state, the electrons of the G-quadruplex occupy the ground state of the fluorophore, and it will be difficult for the electrons of the fluorophore to return to the ground state, resulting in fluorescence-quenching.

### 3.3. G-Quadruplex-Based Label-Free and Quencher-Free Fluorescence Detection Platform for OTA and K^+^

**Figure 3:**
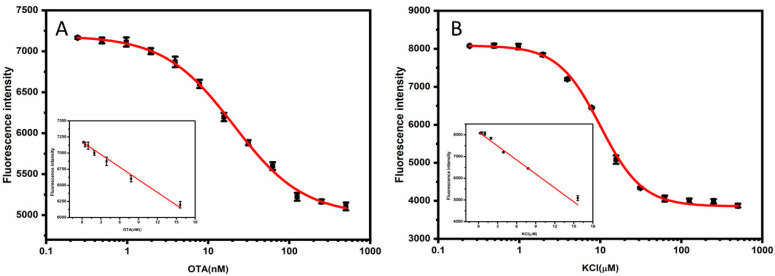
(**A**) Calibration curve for OTA. The inset shows the linear relationship between the fluorescence intensity and the concentration of the OTA (**B**) Calibration curve for K^+^. The inset shows the linear relationship between the fluorescence intensity and the concentration of K^+^. The error bar represents the standard deviations of the three parallel experiments.

Based on the quenching effect of G-quadruplex on the fluorophore, we constructed an ultrasensitive detection platform with a label-free fluorophore and quencher, with no complementary strands. The sensitive detection platform allows the highly sensitive determination of OTA and K^+^. Considering the simplicity and practicality of the detection process, we achieved the detection of OTA and K^+^ using an antiparallel G-quadruplex (OTAapt31) and parallel G-quadruplex (5AG4). The results are shown in Figure 3.

The linear range of the OTA sensor was from 0.19 to 15.63 nM, and the limit of detection (LOD) was 0.19 nM; the linear equation was F = 7174.24 − 65.42 *C*_OTA_ (nM), with a linear correlation coefficient of 0.987. The linear range of the K^+^ sensor was from 0.24 to 15.63 µM, and the LOD was 0.24 µM; the linear equation was F = 8130.76 − 214.47 *C*_K_^+^ (µM), with a linear correlation coefficient of 0.996. The performance of our bioassay was compared with previously reported sensors. As shown in Table S2, the detection performance of our method was comparable to that reported in most of the other studies. Our method adopts a non-competitive strategy, so the detection limit is one order of magnitude lower than that of the competitive strategy [22] and comparable to methods of enzymatic amplification strategies [30,31]. Therefore, it can be concluded that our assay technique may provide a simple, fast, and cost-effective method for sensitive quantification.

### 3.4. Specificity of the Aptasensor

The selectivity of the sensing method was evaluated by monitoring the fluorescence response of the sensing system in the presence of common analogs, metal ions, or toxins. For the antiparallel G-quadruplex system, we replace OTA with OTB, AFB1, AFG1, FB1, Patulin, ZEA, and DON; for the parallel G-quadruplex system, we replace K^+^ with NH_4_^+^, Na^+^, Ca^2+^, Mg^2+^, Zn^2+^, Cd^2+^.

**Figure 4:**
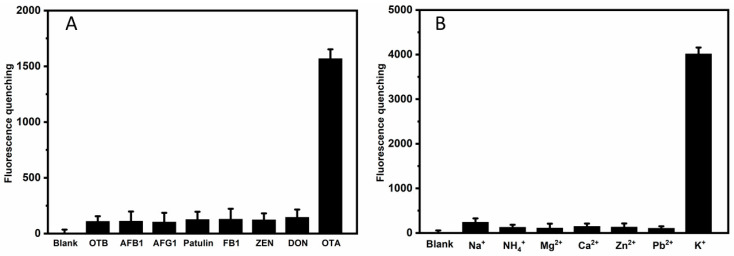
(**A**) Selectivity performance of sensing system for OTA detection with the addition of OTB, AFB1, AFG1, Patulin, FB1, ZEN, and DON to the solution. The concentration of ions is 62.5 nM. (**B**) Selectivity performance of sensing system for K^+^ detection with the addition of NH_4_^+^, Na^+^, Ca^2+^, Mg^2+^, Zn^2+^, and Cd^2+^ions to the solution. The concentration of toxins is 62.5 µM. The fluorescence-quenching is the difference between response and blank. The error bar represents the standard deviations of the three parallel experiments.

As shown in Figure 4A, the sensor has no apparent response to OTB, AFB1, AFG1, FB1, patulin, ZEA, and DON, demonstrating the specificity of the bioassay. The results in Figure 4B also show that compared with K^+^, other metal ions cannot produce strong fluorescence response signal changes, proving that this fluorescence detection platform has high selectivity for K^+^ sensing. The parallel G-quadruplex has a channel in its center with a diameter close to the ionic radius of K^+^ (1.3 Å) [32]. By binding to the eight carbonyl oxygen atoms of the G-quadruplex, K^+^ can be located in the cavity between two adjacent G-tetrads of the G-quadruplex. Of all the alkali metal cations, this coordination contributes to the highest efficiency of K^+^ in stabilizing the G-quadruplex, which confers selectivity of the G-quadruplex DNA for K^+^ [33].

### 3.5. Practical Application

To evaluate the practical application of the proposed fluorescence system, the method was applied in the detection of OTA in red wine and K^+^ in sheep serum, respectively. The standard addition recovery experiment determined the OTA in the red wine sample. The spiked samples were pretreated using the extraction protocol described in our previous study [21]. The samples after pretreatment were detected according to the method described in Section 2.4, and the fluorescence intensity was recorded. As shown in Table 1, the standard addition recovery rate of different concentrations of OTA was between 97.75% and 103.25%, showing that the method was reliable for detecting OTA in the red wine samples.

Goat serum samples were diluted 1000-fold with Tris-HCl buffer, and then the K^+^ concentration was calculated according to the linear correction equation. The results showed that the K^+^ concentration measured by this method was 6.54 ± 0.41 mmol/L (RSD = 2.1%, *n* = 5). The goat serum sample was determined by atomic absorption spectrometry and the K^+^ concentration obtained was 6.41 ± 0.54 mmol/L (RSD = 2.8%, *n* = 5). Comparing the two sets of data, we can conclude that the measured values are in good agreement with the standard values, showing that this method can be used to detect K^+^ concentration in serum.

## 4. Conclusions

In this study, we demonstrate that the fluorescence-quenching effect of G-quadruplex is based on the PIET principle. We introduced specific ligands for G-quadruplex leading to a further quenching and confirmed the mechanism of fluorescence-quenching of G-quadruplex based on PIET. A simple label-free fluorophore and quencher fluorescence platform was established and applied to detect OTA and K^+^ based on the high fluorescence-quenching ability of G-quadruplexes. In the presence of OTA or K^+^, the nucleic acid probe folds to form a G-quadruplex structure, leading to the fluorescence-quenching of the fluorophore. Besides the high sensitivity, the sensing system exhibits good selectivity and practical detection capability. Our method adopts a non-competitive strategy, so the detection limits were lower than that of the competitive strategy and even comparable to some methods of enzymatic amplification strategies. Consequently, G-quadruplexes can be excellent candidates as quenchers, and the strategy implemented in the study can be extended to an aptasensor with G-quadruplex.

## Figures and Tables

**Table 1:** Recovery experiments of OTA determination in red wine samples.

Sample Number	Added (nM)	Detected (nM)	Recovery (%)
1	2.00	1.98 ± 0.53	99.00
2	4.00	4.13 ± 0.35	103.25
3	8.00	7.82 ± 0.62	97.75

## Data Availability

Not applicable.

## Supplementary Materials

The following supporting information can be downloaded at: https://www.mdpi.com/article/10.3390/bios12060423/s1, Figure S1: Optimization of the SGI and Ca^2+^ concentration; Figure S2: Optimization of Ca^2+^ and the complementary sequence (A14) concentration; Figure S3: (A) Fluorescence emission spectra of FAM labeled fluorescence antiparallel G-quadruplex with different concentrations of OTA, (B) Fluorescence emission spectra of FAM labeled fluorescence parallel G-quadruplex with different concentrations of K^+^; Figure S4: Fluorescence response curve of OTAapt31-FAM with different complementary sequence and different concentrations of OTA; Figure S5: Fluorescence response curve of different sequences of parallel G-quadruplexes (unlabeled) under different concentrations of K^+^; Table S1: Sequences of the different constructs used in this study and their IC_50_; Table S2: Comparison of different methods for OTA and K^+^ Determination.
