# Supplementary material for: Development of Fluorescent Aptasensors Based on G-Quadruplex Quenching Ability for Ochratoxin A and Potassium Ions Detection
Source: Biosensors (Basel). 2022 Jun 16;12(6):423. doi: 10.3390/bios12060423 (PMC9221108; doi:10.3390/bios12060423)
Supplement: Supplementary file 1 [file biosensors-12-00423-s001.zip › biosensors-1733815-supplementary.pdf]

biosensors

Supplmentary

Development of Fluorescent Aptasensors Based on G-Quadruplex Quenching Ability for Ochratoxin A and Potassium Ions Detection

Cheng Yang [1,*], Xiaolin Chu [1], Li Zeng [2], Amina Rhouati [3], Fathimath Abbas [1], Shengnan Cui [1,] and Daiqin Lin [2,*]

[1] State Key Laboratory of Fine Chemicals, Department of Chemistry, School of Chemical Engineering, Dalian University of Technology, Dalian 116024, China; 21907036@mail.dlut.edu.cn (X.C.); ferthunabbas@mail.dlut.edu.cn (F.A.); cuishengnan@mail.dlut.edu.cn (S.C.)
[2] Institute of Inspection and Testing for Industrial Products of Jiangxi General Institute of Testing, Nanchang 330052, China; zengli19890610@163.com
[3] Bioengineering Laboratory, Higher National School of Biotechnology, Constantine 25100, Algeria; amina.rhouati@gmail.com
* Correspondence: author, Email: yangcheng@dlut.edu.cn (C.Y.); zzl0711@126.com (D.L.)

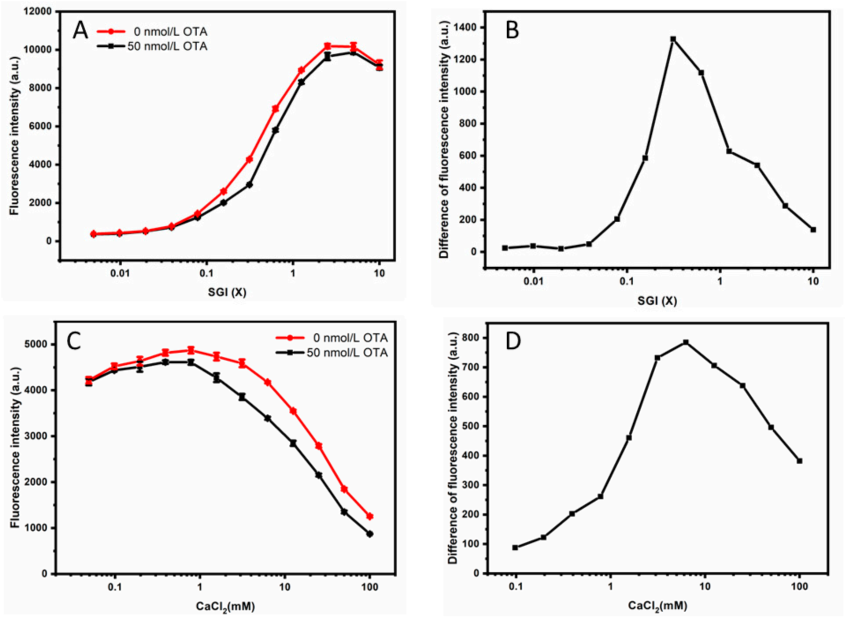
A
0 nmol/L OTA
50 nmol/L OTA
Fluorescence intensity (a.u.)
SGI (X)
B
Difference of fluorescence intensity (a.u.)
SGI (X)
C
0 nmol/L OTA
50 nmol/L OTA
Fluorescence intensity (a.u.)
CaCl2(mM)
D
Difference of fluorescence intensity (a.u.)
CaCl2(mM)

Figure S1. Optimization of the SGI and Ca^{2+} concentration. (A)Fluorescence intensity of different concentrations of SGI in the absence and presence of OTA. (B). The difference in fluorescence intensity from Figure S1A. (C). Fluorescence intensity for different concentrations of Ca^{2+} in the absence and presence of OTA. (D). The difference in fluorescence intensity from Figure S1C. OTAapt31 aptamer, and OTA concentration was 20 nmol/L and 50 nmol/L, respectively. The standard deviations of the three parallel experiments were indicated by error bars.

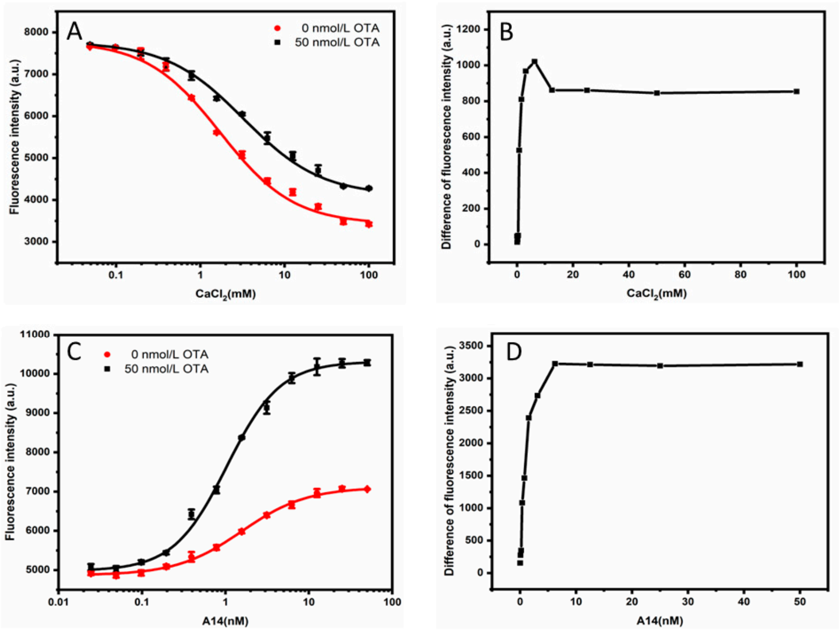

Figure S2. Optimization of Ca^{2+} and the complementary sequence (A14) concentration. (A). Fluorescence intensity of different concentrations of Ca^{2+} in the absence and presence of OTA. (B). The difference in fluorescence intensity from Figure S2A. (C). Fluorescence intensity for different concentrations of A14 in the absence and presence of OTA. (D). The difference in fluorescence intensity from Figure S2C. OTAapt31-FAM aptamer, and OTA concentration was 20 nmol/L and 50 nmol/L, respectively. The standard deviations of the three parallel experiments were indicated by error bars.

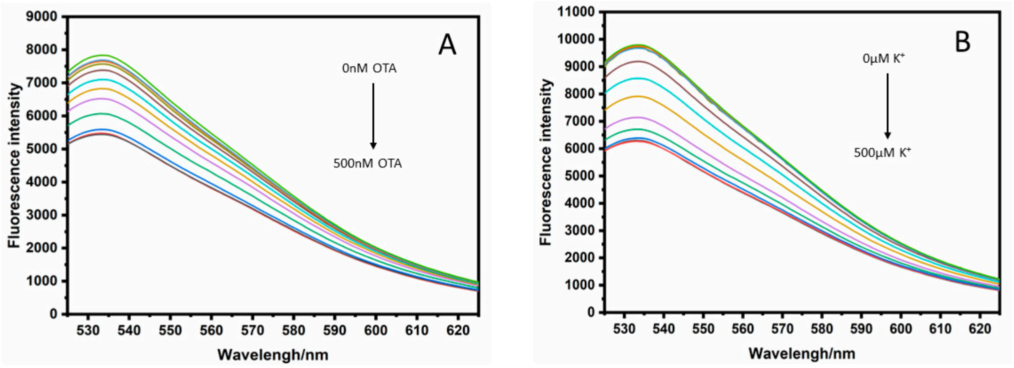

Figure S3 (A) Fluorescence emission spectra of FAM labeled fluorescence antiparallel G-quadruplex with different concentrations of OTA (0, 0.48, 0.96, 1.95, 3.9, 7.8,15.6, 31.2, 72.5, 125, 250, 500 nM), (B)Fluorescence emission spectra of FAM labeled fluorescence parallel G-quadruplex with different concentrations of K^+ (0, 0.48, 0.96, 1.95, 3.9, 7.8,15.6, 31.2, 72.5, 125, 250, 500 μM). The concentration of 5AG4 aptamer was 20 nM.

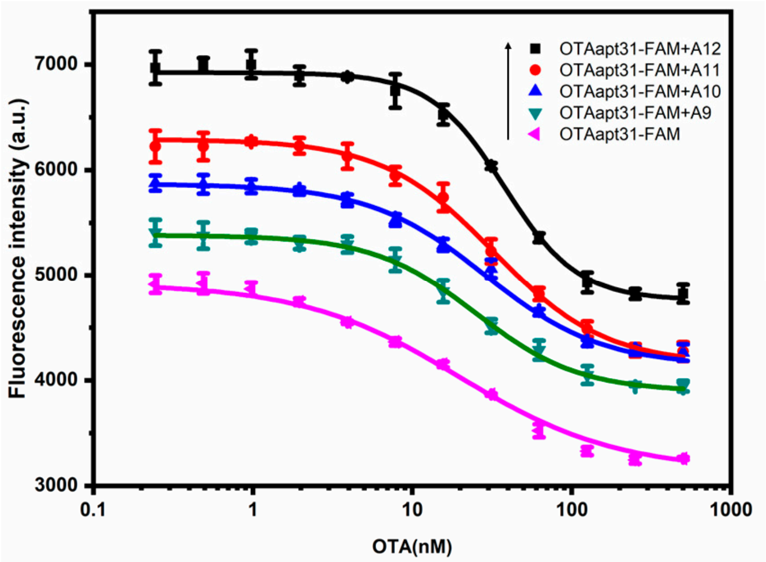

Figure S4 Fluorescence response curve of OTAapt31-FAM with different complementary sequence and different concentrations of OTA.

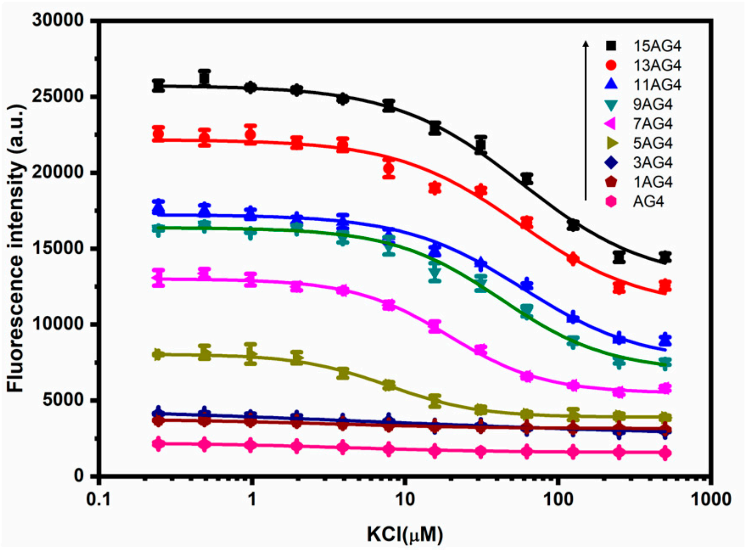

Figure S5 Fluorescence response curve of different sequences of parallel G-quadruplexes (unlabeled) under different concentrations of K^{+}. The concentration of 5AG4 aptamer was 20 nM The standard deviations of the three parallel experiments were indicated by error bars.

Table S1 Sequences of the different constructs used in this study and their IC_{50}

ID	Base sequence	IC_{50} (nM)
G4	GGGTGGGTGGGTGGG	3.2
1AG4	AGGGTGGGTGGGTGGGT	4.1
3AG4	AAAGGGTGGGTGGGTGGGATTT	5.3
5AG4	AAAAAGGGTGGGTGGGTGGGTTTTT	7.8
5AG4-FAM	AAAAAGGGTGGGTGGGTGGGTTTTT-FAM	8.1
7AG4	AAAAAAAGGGTGGGTGGGTGGGTTTTTTT	19.4
9AG4	AAAAAAAAAGGGTGGGTGGGTGGGTTTTTTTTT	42.7
11AG4	AAAAAAAAAAAGGGTGGGTGGGTGGGTTTTTTTTTTT	58.1
13AG4	AAAAAAAAAAAAAGGGTGGGTGGGTGGGTTTTTTTTTTTTT	58.6
15AG4	AAAAAAAAAAAAAAAGGGTGGGTGGGTGGGTTTTTTTTTTTTTTT	58.9
G4-C11	CCCACCCACCC	-
G4-C13	CCCACCCACCCAC	-
G4-C15	CCCACCCACCCACCC	-
OTAapt36	GATCGGGTGTGGGTGGCGTAAAGGGAGCATCGGACA	55.7
OTAapt31	GATCGGGTGTGGGTGGCGTAAAGGGAGCATC	20.6
OTAapt31-FAM	GATCGGGTGTGGGTGGCGTAAAGGGAGCATC-FAM	19.8
G31C	GGATCGGGTGTGGGTGGCGTAAAGGGAGCATCC	17.8
OTA-A9	ATGCTCCCT	-
OTA-A10	ATGCTCCCTT	-
OTA-A11	ATGCTCCCTTT	-
OTA-A12	ATGCTCCCTTTA	-

The underlined portion can form a self-hybridizing duplex strand.

Table S2 Comparison of different methods for OTA and K^+ Determination

Method	Principle	Target	Linear range	LOD	Ref.
Fluorescence	Graphene oxide (GO) and detachable nanoladders based biosensors	OTA	50-80nM	4.59 nM	[1]
Fluorescence	GQ-to-toxin energy transfer-based aptasensor	OTA	15-73.25nM	5 nM	[2]
Chemiluminescence	Magnetic microsphere (MBs) separated carrier-based aptasensor	OTA	0.25-50nM	0.1nM	[3]
Chemiluminescence	Forster resonance energy transfer (FRET)	OTA	0.25-250nM	0.55 nM	[4]
Colorimetry	G-quadruplex-hemin DNAzyme gold nanorods (AuNRs) mediated aptasensor	OTA	10-200nM	10nM	[5]
Fluorescence	G-quadruplex quenching ability used for quenching fluorescence	OTA	0.19-15.63nM	0.19nM	This work
Fluorescence	G-quadruplex ligand fluorescence-based aptasensor	K^+	0.5-10mM	0.5mM	[6]
Fluorescence	mitochondria-targeted fluorescent sensor	K^+	0.5-5mM	0.5mM	[7]
Fluorescence	GQDs modified ionophore-based optical nanosensors	K^+	3-1000μM	3μM	[8]
Chemiluminescence	a guanine chemiluminescence (CL) system	K^+	30-1000μM	30nM	[9]
Colorimetry	crown ether modified Au NPs based sensor	K^+	0-200μM	5.24μM	[10]
Fluorescence	G-quadruplex quenching ability used for quenching fluorescence	K^+	0.24-15.63μM	0.24μM	This work

References:

1. Shao, X. L.; Zhu, L. J.; Feng, Y. X.; Zhang, Y. Z.; Luo, Y. B.; Huang, K. L.; Xu, W. T., Detachable nanoladders: A new method for signal identification and their application in the detection of ochratoxin A (OTA). *Anal. Chim. Acta* **2019**, 1087, 113-120.doi:10.1016/j.aca.2019.08.057
2. Armstrong-Price, D. E.; Deore, P. S.; Manderville, R. A., Intrinsic “Turn-On” aptasensor detection of ochratoxin A using energy-transfer fluorescence. *J. Agr. Food Chem.* **2020**, 68, 2249-2255.doi:10.1021/acs.jafc.9b07391
3. Yan, X. L.; Xue, X. X.; Luo, J.; Jian, Y. T.; Tong, L.; Zheng, X. J., Construction of chemiluminescence aptasensor platform using magnetic microsphere for ochratoxin A detection based on G bases derivative reaction and Au NPs catalyzing luminol system. *Sensors and Actuators B: Chemical* **2020**, 320, 128375.doi:10.1016/j.snb.2020.128375
4. Su, R. F.; Zheng, H. R.; Dong, S. Y.; Sun, R.; Qiao, S. N.; Sun, H. J.; Ma, X. Y.; Zhang, T. H.; Sun, C. Y., Facile detection of

melamine by a FAM‑aptamer‑G-quadruplex construct. *Anal. Bioanal. Chem.* **2019**, 411, 2521-2530.doi:10.1007/s00216-019-01688-3

5. Yu, X. H.; Lin, Y. H.; Wang, X. S.; Xu, L. J.; Wang, Z. W.; Fu, F. F., Exonuclease-assisted multicolor aptasensor for visual detection of ochratoxin A based on G-quadruplex-hemin DNAzyme-mediated etching of gold nanorod. *Microchim. Acta* **2018**, 185.doi:10.1007/s00604-018-2811-9
6. Yang, L.; Qing, Z. H.; Liu, C. H.; Tang, Q.; Li, J. S.; Yang, S.; Zheng, J.; Yang, R. H.; Tan, W. H., Direct fluorescent detection of blood potassium by ion-selective formation of intermolecular G-Quadruplex and ligand binding. *Anal. Chem.* **2016**, 88, 9285-9292.doi:10.1021/acs.analchem.6b02667
7. Ning, J. W.; Tian, Y. Q., Development of a new simple mitochondria-targeted fluorescent K^+ sensor and the application in high-throughput monitoring K^+ fluxes. *Sensors and Actuators B: Chemical* **2020**, 307, 127659.doi:https://doi.org/10.1016/j.snb.2020.127659
8. Wang, R. J.; Du, X. F.; Wu, Y. T.; Zhai, J. Y.; Xie, X. J., Graphene quantum dots integrated in ionophore-based fluorescent nanosensors for Na^+ and K^+. *ACS Sensors* **2018**, 3, 2408-2414.doi:10.1021/acssensors.8b00918
9. Dong, J. J.; Zhao, H. Z.; Zhou, F. L.; Li, B. X., Rapid and sensitive detection of potassium ion based on K^+-induced G-quadruplex and guanine chemiluminescence. *Anal. Bioanal. Chem.* **2016**, 408, 1863-1869.doi:10.1007/s00216-015-9285-y
10. Qiu, J. Y.; Zhang, Y. J.; Dong, C.; Huang, Y. L.; Sun, L.; Ruan, H. M.; Wang, H. S.; Li, X.; Wu, A. G., Rapid colorimetric detection of potassium ions based on crown ether modified Au NPs sensor. *Sensors and actuators. B, Chemical* **2019**, 281, 783-788.doi:10.1016/j.snb.2018.10.139
